# NfL and GFAP quantification and associations with mental health in recreational cannabis users—Results from the Swiss study on recreational cannabis access via pharmacies

**DOI:** 10.1007/s12035-026-05798-9

**Published:** 2026-03-13

**Authors:** Maximilian Meyer, C. Felix Mosandl, Adrian Guessoum, Oliver Herrmann, Lavinia Baltes-Flückiger, Eva-Maria Pichler, Amar Zadic, Aleksandra Maleska Maceski, Juan Francisco Vilchez Gomez, Kenneth M. Dürsteler, Felix Müller, Undine E. Lang, Pascal Benkert, Jens Kuhle, Marc Walter, Marc Vogel

**Affiliations:** 1 University of Basel Psychiatric Clinics, University of Basel, Basel, Switzerland; 2 Psychiatric Services Aargau, Windisch, Switzerland; 3 Multiple Sclerosis Centre and Research Center for Clinical Neuroimmunology and Neuroscience (RC2NB), Departments of Clinical Research, Neurology and Biomedicine, University Hospital and University of Basel, Basel, Switzerland

**Keywords:** NfL, Neurofilament light chain, GFAP, Glial fibrillary acidic protein, Cannabis, Neuroaxonal damage

## Abstract

Neurofilament light chain (NfL) and glial fibrillary acidic protein (GFAP) are biomarkers for neuroaxonal damage and astroglial injury, respectively. The present study aimed to quantify NfL and GFAP as well as investigate their association with psychiatric symptoms in a sample of recreational cannabis users. Blood samples from 331 individuals with regular recreational cannabis use were collected. Serum NfL (sNfL) and GFAP (sGFAP) levels were quantified in duplicate by an ultrasensitive single-molecule array (Simoa) technology. Reference populations were used to calculate age-, BMI-, and (for GFAP) sex-adjusted *Z* scores. Participants completed validated psychometric instruments assessing symptoms of cannabis use disorder, alcohol use disorder, psychosis, depression, and anxiety. Mean GFAP levels were significantly lower than those in the reference population (*t*(330) = −6.718, *p* < 0.001), whereas NfL levels did not differ (*t*(330) = 1.3, *p* = 0.19). Participants with hazardous cannabis use and those screening positive for psychotic symptoms showed higher NfL levels. Psychotic symptoms were additionally associated with lower GFAP levels. NfL correlated positively with total Cannabis Use Disorder Identification Test–Revised (CUDIT-R) scores (*ρ*(329) = 0.12, *p* = 0.03) and specifically with the items “memory/concentration problems” (*ρ*(329) = 0.11, *p* = 0.04) and “time spent buying/using/recovering” (*ρ*(329) = 0.13, *p* = 0.02). In multiple regression analysis, psychotic and cannabis use disorder symptoms independently predicted higher NfL levels. No variables predicted GFAP levels. There was no association between self-reported cannabis use amount or frequency in the past 30 days and GFAP or NfL. The results provide preliminary evidence of cannabis-related neuroaxonal alterations, warranting longitudinal studies to clarify the temporal relationship between these biomarkers and cannabis use.

**Supplementary Information:**

The online version contains supplementary material available at https://doi.org/10.1007/s12035-026-05798-9.

## Introduction

Recreational cannabis use is associated with the development of depression, anxiety, psychosis, and cannabis use disorder (CUD) [1, 2]. The risk for developing psychosis and CUD increases with cannabis potency, i.e. the concentration of Δ^9^-tetrahydrocannabinol (THC) in the product [3]. Furthermore, the risk of developing mental illness is associated with the frequency of use, duration of use, and initiation of use in early adolescence [4].

Some studies found a link between heavy cannabis use and impairment of cognitive functions. Acute effects comprise reduced executive functions such as concentration, decision-making, and working memory [5]. Evidence on long-term effects on cognition is equivocal [6]. Reductions of neuropsychological functioning have been observed in users with heavy and frequent use patterns, although in some studies this link was no longer present after controlling for confounding variables [7]. In a large cohort study, long-term cannabis users were followed up from childhood to midlife. They showed a mean IQ decline of 5.5, a decline in learning and processing speed, memory and attention deficits, and smaller hippocampal volume compared to nonusers [8]. However, evidence on cannabis effects on hippocampal volume is particularly inconsistent in current literature with other studies observing no effect [9, 10].

No consensus exists regarding the underlying mechanism linking cannabis use to cognitive impairment. However, Bourque and Potvin describe three important hypotheses [11]. The cognitive vulnerability hypothesis states that preexisting cognitive deficits may put individuals at higher risk of becoming regular users. The concurrent hypothesis states that only short-term associations between cannabis use and cognitive impairment exist, when controlling for premorbid cognitive function. Finally, the neurotoxicity hypothesis proposes that long-term cognitive impairment following cannabis use persists, even when cannabis use is stopped.

Biomarkers such as neurofilament light chain (NfL) and glial fibrillary acidic protein (GFAP) have recently gained attention for their predictive role in multiple sclerosis disease activity and progression [12]. Neurofilaments are scaffolding proteins that are exclusively expressed in neurons [13]. Serum NfL (sNfL) levels have been associated with neuronal cell damage, whereas serum GFAP (sGFAP) levels are associated with astroglial injury. Furthermore, elevated sNfL and sGFAP levels have been associated with brain volume loss and cognitive decline [14–16].

Recent literature underlined the significant potential of NfL as a biological marker for diagnostic algorithms and treatment response monitoring in psychiatric disorders [17, 18]. However, although NfL elevations are found in patients with mood disorders, early-stage psychotic disorders, and substance use disorders, NfL levels vary across diagnostic entities and interindividual factors, most importantly age and to a lesser extent body mass index (BMI) [19, 20].

Conversely, the association between psychiatric disorders and GFAP levels has been inconclusive. A recent study found lower sGFAP levels in patients with unipolar depression compared to healthy controls, but no association with depression severity was found [21], whereas Steinacker et al. found increased sGFAP levels in patients with depression [22]. Overall, the relationship between sGFAP and psychiatric disorders is unclear, whereas elevated sNfL levels are associated with a range of psychiatric disorders and neurological diseases.

Although for both GFAP and NfL group differences were found when comparing psychiatric disorder groups to healthy controls, there is a lack of evidence for associations between NfL/GFAP levels and symptom severity. Hviid et al. found no association between these biomarkers and depression scores in a sample of treatment-naïve patients with unipolar depression [21]. For psychotic disorders, to our knowledge, no literature exists on a potential link between symptom severity specifically and NfL/GFAP levels.

In substance use disorders specifically, a positive association between cocaine use and NfL plasma levels has been observed [23]. The authors discuss microstructural adaptations following neurometabolic dysregulations through repeated cocaine use as a potential mechanism of peripheral NfL elevation. Li et al. found a 2.53-fold increase of sNfL in patients with alcohol use disorder when compared with healthy controls and conclude that white matter lesions associated with alcohol use lead to increased peripheral NfL concentrations [24].

Literature on the association between cannabis use and neurofilaments is scarce. Although brain-imaging studies point towards an alteration of glial function in long-term cannabis users, no studies investigating the impact of cannabis use on NfL or GFAP levels in humans exist to date [25]. Recent studies on substance use and major depressive disorder proposed the altered activity of neurotrophic factors as a potential underlying mechanism of increased brain vulnerability that may also be relevant in cannabis use [26, 27]. However, the neurotoxic properties of cannabis are likely less pronounced when compared to alcohol or cocaine [28]. Furthermore, research investigating structural or physiological changes following cannabis exposure was mostly conducted in animal models [29].

The present study investigated the relationship between sNfL as well as sGFAP levels and mental health (symptoms of depression, anxiety, psychosis, attention deficit and hyperactivity disorder (ADHD), and CUD) in a cohort of individuals reporting regular nonmedical cannabis use while controlling for other substances known to alter NfL/GFAP levels. Specifically, in line with the neurotoxicity hypothesis, we tested whether associations between neurofilament levels and CUD symptoms as assessed by validated instruments exist.

## Methods and materials

### Participants

Blood samples were collected from participants of the WeedCare study (NCT05522205). WeedCare investigates the impact of regulated, pharmacy-based access to recreational cannabis products for regular nonmedical cannabis users in Basel, Switzerland [30]. Inclusion criteria comprised the minimum age of 18, cannabis use at least once per month during the past 6 months, a THC-positive urine sample, sufficient German language skills, current residence in the canton Basel-Stadt, and internet access. Exclusion criteria were current pregnancy or breastfeeding, current psychiatric inpatient hospital stay, acute state of psychosis or suicidality, severe cognitive impairment limiting the ability to provide written informed consent or understand study procedures, and the intention to move away from the canton Basel-Stadt. A total of 331 participants agreed to provide a blood sample and their biological sex and completed the baseline WeedCare questionnaires. Because the study started with a slight delay, there was a time period of up to 16 weeks between blood sampling and subsequent questionnaire assessment in some cases.

### Assessments

Assessment of sociodemographic and anthropometric data included age, sex, weight, height, and education. Additionally, substance use in the past six months was assessed.

#### Cannabis Use

The Cannabis Use Disorder Identification Test–Revised (CUDIT-R) was employed. The CUDIT-R consists of eight items, rated on a 5-point Likert scale ranging from 0 to 4, with a score of 8 or higher indicating hazardous use and a score of 13 or higher indicating possible CUD (sensitivity: 0.913 and specificity: 0.900) [31].

#### Psychometric instruments

Anxiety symptoms were assessed with the Generalized Anxiety Disorder Scale-7 (GAD-7), which consists of seven items rated on a 4-point Likert scale, with a score of 10 or higher being indicative for current generalised anxiety disorder (sensitivity: 0.89 and specificity: 0.82) [32].

Depression symptoms were assessed with the Patient Health Questionnaire for Depression (PHQ-9), consisting of nine items rated on a 4-point Likert scale. A cutoff score of 10 or higher indicates major depression (sensitivity: 0.88, specificity: 0.88) [33].

Psychotic symptoms were assessed with the Early Recognition Inventory (ERIraos) [34]. Only items screening for early psychosis risk were included. These eight items are answered with either yes (1) or no (0), with a total score of 1 or higher being indicative for early psychosis.

ADHD symptoms were screened for with the Adult ADHD Self-Report Scale (ASRS-V1.1), consisting of six items rated on a 5-point Likert scale, with each item requiring either three or four scale points to fulfil the criterion for the corresponding symptom. A total symptom score of 4 or more is considered a positive screening result for adult ADHD (sensitivity 0.69 and specificity 1) [35].

Alcohol use disorder was assessed with the Alcohol Use Disorder Identification Test–Consumption (AUDIT-C) [36]. Three items are rated on a 5-point Likert scale to create a sum score ranging from 0 to 12. A score of 4 or higher in men and 3 or higher in women is indicative of alcohol use disorder in the European population (sensitivity 0.63 and specificity 0.72 for men and sensitivity 0.76 and specificity 0.79 for women) [37].

### NfL and GFAP measurements

Blood samples were collected at study inclusion between September 2022 and March 2023. The serum tubes (S-Monovette® Serum CAT, 7.5 ml) were centrifuged for 10 min at 4 °C and serum aliquots were stored at −80 °C.

Serum NfL and GFAP were measured using the single molecule array (SIMOA) Neurology 2-plex B assay (Quanterix, Billerica, MA) on the HDx platform, in accordance with the manufacturer’s instructions. Samples were measured in duplicate, and few samples exceeding the intra-assay coefficient of variation (CV) of 20% were reanalysed. NfL and GFAP concentrations were within the assay measurement range. Three internal serum quality controls (QCs) with low, medium, and high NfL and GFAP concentrations were included in each run. The mean inter-assay CV of these QCs was 3.5%, 2.3%, and 4.5% for NfL (mean concentration of 8.7 pg/ml, 14.3 pg/ml, and 105.7 pg/ml, respectively) and 6.7%, 3.2%, and 3.2% for GFAP (mean concentration of 75.3 pg/ml, 147.8 pg/ml, and 484.6 pg/ml, respectively).

Due to NfL’s and GFAP’s dependence on age, sex, and BMI, adjusted *Z* scores were calculated for further analyses. The reference database for NfL was derived from participants of four large population-based studies and control groups of multiple sclerosis studies (*n* = 5390). The age of included persons ranged from 18.0 to 81.0 years old [19]. The GFAP reference database was compiled from three cohorts (*n* = 4297). The age of included persons ranged from 35.5 to 53.9 years old, mean BMI was 25.4, and 54.9% were female [38]. Individuals in these reference databases did not have documented neurological disease, and the same assays were used for this study and the reference populations [19, 38].

### Statistical analyses

All analyses were done with SPSS v29 (IBM) and R version 4.5.1 (http://www.r-project.org). Kolmogorov–Smirnov and Shapiro–Wilk tests as well as visual assessments were used to assess data distribution. All calculations were based on *Z* scores. One-sample *t* tests and Cohen’s *d* were calculated to compare the distributions of neurofilament *Z* scores to standard normal distribution (*Z* score = 0). Welch’s *t* tests were used to compare groups defined by psychometric instrument cutoffs (below vs. at cutoff and above), and Hedges’ *g* was calculated as a measure of effect size. Spearman’s *ρ* was calculated to test for associations between continuous variables (i.e. psychometric instrument scores and sNfL/sGFAP levels) when they were not normally distributed. Pearson’s *r* was calculated to test for associations between self-reported cannabis use and sNfL/sGFAP levels. Multiple linear regression models were calculated to quantify the influence of other variables (i.e. age, sex, and psychometric instrument scores) on sNfL and sGFAP levels. sNfL and sGFAP *Z* scores account for age- and sex-related variation in normative biomarker levels. However, age and sex were additionally included as covariates in the regression models, as they may independently confound the relationship between cannabis use variables and biomarker outcomes beyond their influence on normative biomarker distributions. All variables which were significantly associated with sNfL or sGFAP, respectively, were entered in the model. Furthermore, age and sex were entered due to their general relevance in biomarker research. For sNfL, alcohol, cocaine, and opioid use were entered as binary confounders due to their reported association with altered NfL levels [23, 24, 39]. Variance inflation factors (VIFs) were calculated to assess multicollinearity. Level of significance was set at *p* < 0.05 for all analyses.

## Results

### Sociodemographic and medical characteristics

Sociodemographic and medical characteristics of participants are summarised in Table 1.

**Table 1:** Sociodemographic and medical characteristics of the sample (*n* = 331)

		*M* (SD)	*n* (%)
Age		35.5 (11.6)	
Sex	Male		271 (81.9)
	Female		60 (18.1)
Education	Compulsory education		31 (9.4)
	Vocational education		93 (28.1)
	General secondary education (university entrance diploma)		54 (16.3)
	Higher vocational education		24 (7.3)
	University		125 (37.8)
	Other		4 (1.2)
Substance use in the past 6 months	Alcohol		300 (90.6)
	Hallucinogens		68 (20.5)
	Amphetamines		68 (20.5)
	Cocaine		67 (20.2)
	Benzodiazepines		31 (9.4)
	Opioids		15 (4.5)
	Glue/solvent		7 (2.1)
Cannabis use in the past 30 days (*n* = 329)	CBD flowers		118 (35.6)
	CBD hashish		25 (7.6)
	CBD concentrate^a^		24 (7.3)
	THC flowers		302 (91.2)
	THC hashish		194 (58.6)
	THC concentrate		54 (16.3)
Days of THC cannabis use in the past 30 days (*n* = 320)		18.2 (11.0)	
Average amount of THC cannabis used per day in grammes (*n* = 321)		1.4 (2.6)	

*M* mean, *SD* standard deviation, *CBD* cannabidiol, *THC* Δ^9^-tetrahydrocannabinol

^a^CBD products such as oils and crystals are legally available for commercial sale in Switzerland

### Psychometric Characterisation

Psychometric assessments are summarised in Table 2. Sample characteristics by CUDIT-R group are provided in the Supplemental Material (Table A1).

**Table 2:** Psychometric instrument scores (*n* = 331)

Instrument		*M* (SD)	*n* (%) above cutoff
CUDIT-R		10.9 (4.5)	
	Hazardous use^a^		253 (76.4)
	Possible CUD^b^		107 (32.3)
PHQ-9		5.1 (3.6)	36 (10.9)
GAD-7		3.6 (3.2)	16 (4.8)
ASRS		1.6 (1.5)	44 (13.3)
ERIraos		0.5 (0.9)	106 (32)
AUDIT-C		3.8 (2.5)	134 (40.5)

*M* mean, *SD* standard deviation, *CUDIT-R* Cannabis Use Disorder Identification Test–Revised, *PHQ-9* Patient Health Questionnaire for Depression, *GAD-7* Generalized Anxiety Disorder Scale-7, *ASRS* Adult ADHD Self-Report Scale, *ERIraos* Early Recognition Inventory for Psychosis, *AUDIT-C* Alcohol Use Disorder Identification Test–Consumption

^a^CUDIT-R score ≥ 8

^b^CUDIT-R score ≥ 13

*sNfL and sGFAP Levels Compared to the Reference Population*

Participants had lower sGFAP *Z* scores compared to the reference population (*M* = −0.40, *t*(330) =  −6.718, *p* < 0.001, *d* = −0.37). No difference was found for sNfL levels (*M* = 0.07, *t*(330) = 1.3, *p* = 0.19, *d* = 0.07). For sGFAP, the median was −0.50 and the IQR was 1.46. For sNfL, the median was 0.15 and the IQR was 1.38. Neurofilament *Z* score distributions are provided in Fig. 2.

**Fig. 1:**
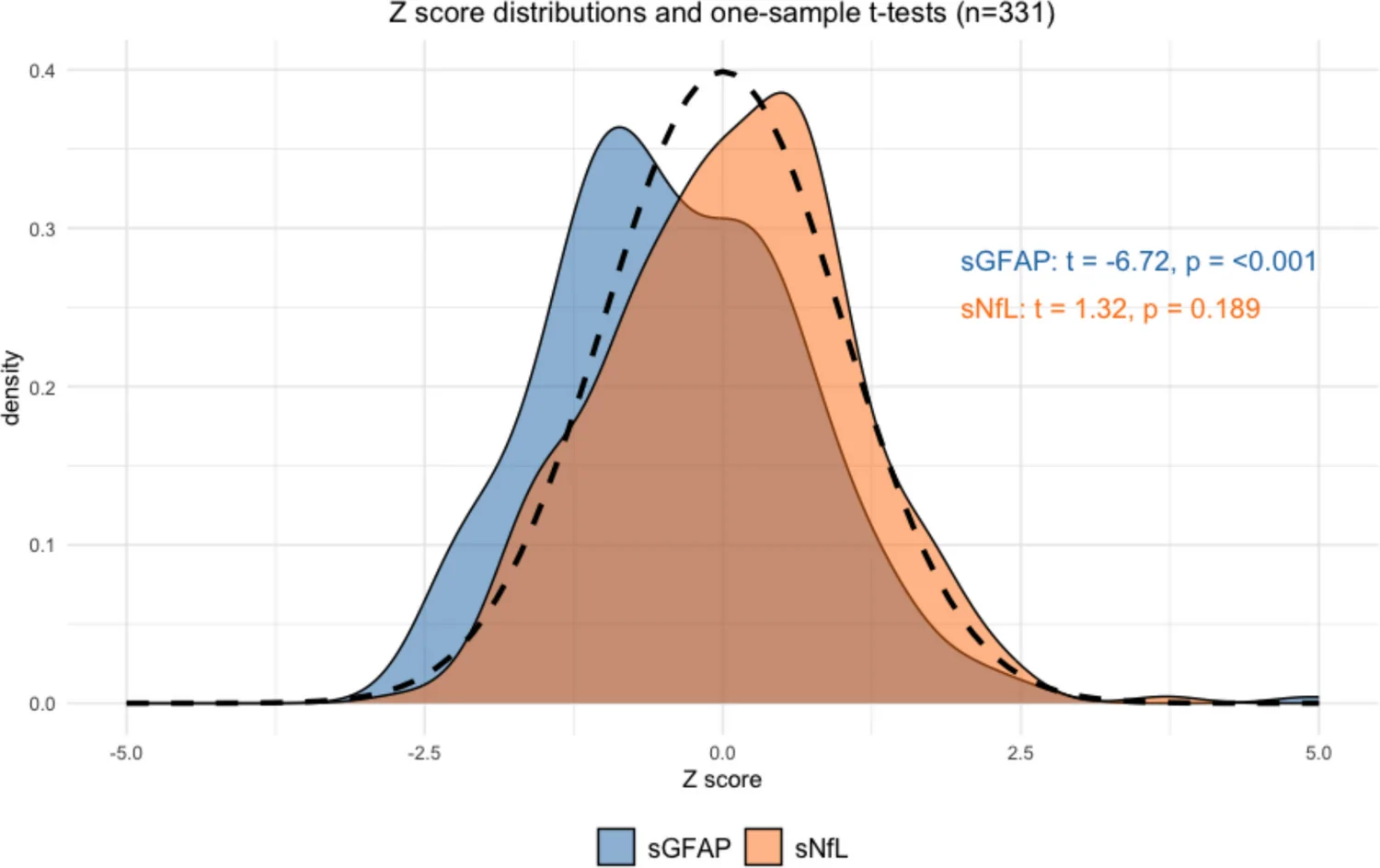
sNfL and sGFP level distributions with reference normal distribution. Dotted: normal distribution. sGFAP, serum glial fibrillary acidic protein; sNfL, serum neurofilament light chain

### Bivariate analyses: comparison of groups by psychometric instrument cutoff

Participants meeting the cutoff of the ERIraos instrument had significantly higher sNfL levels and significantly lower sGFAP levels compared to those below the cutoff. Participants meeting the CUDIT-R cutoff for hazardous use (≥ 8) had significantly higher sNfL levels compared to those below the cutoff, but no difference was found for the groups divided by the CUDIT-R cutoff for possible CUD (≥ 13) (Tables 3 and 4). A linear regression model with CUDIT-R category (below cutoffs, hazardous use, and possible CUD) as the independent variable and sNfL *Z* score as the dependent variable showed that sNfL increased with each higher CUDIT-R category (Table 5). sNfL *Z* score means were −0.15 (SD: 1.02) for the below cutoffs category (*n* = 78), 0.10 (SD: 1.01) for the hazardous use category (*n* = 146), and 0.19 (SD 0.99) for the possible CUD category (*n* = 107).

**Table 3:** Group differences in sNfL *Z* scores by psychometric cutoffs (Welch’s *t* tests, *n* = 331)

Instrument	Participants < cutoff (*M*, SD, *n*)	Participants ≥ cutoff (*M*, SD, *n*)	*t* (df)	*p* (2-sided)	Hedges’ *g* [95% CI]
AUDIT-C	0.12, 1.04, 197	−0.00, 0.97, 134	1.12 (297.6)	0.265	0.12 [−0.10, 0.34]
ERIraos	−0.03, 0.96, 225	0.29, 1.07, 106	−2.57 (187.0)	0.005**	−0.31 [−0.55, −0.08]
PHQ-9	0.08, 1.02, 295	0.05, 0.98, 36	0.13 (44.7)	0.900	0.02 [−0.32, 0.37]
GAD-7	0.06, 1.00, 315	0.34, 1.17, 16	0.94 (16.1)	0.362	−0.28 [−0.78, 0.22]
ASRS	0.04, 1.00, 287	0.31, 1.05, 44	−1.62 (55.6)	0.112	−0.27 [−0.59, 0.05]
CUDIT-R hazardous use	−0.15, 1.02, 78	0.14, 1.00, 253	−2.23 (125.6)	0.028*	−0.29 [−0.55,− 0.04]
CUDIT-R possible CUD	0.01, 1.02, 224	0.19, 0.99, 107	−1.54 (214.7)	0.126	−0.18 [−0.41, 0.05]

*M* mean,* SD* standard deviation, *n* sample size, *CI* confidence interval, *AUDIT-C* Alcohol Use Disorder Identification Test–Consumption, *ERIraos* Early Recognition Inventory for Psychosis, *PHQ-9 *Patient Health Questionnaire for Depression, *GAD-7 *Generalized Anxiety Disorder Scale-7, *ASRS* Adult ADHD Self-Report Scale, *CUD* cannabis use disorder, *CUDIT-R* Cannabis Use Disorder Identification Test–Revised (hazardous use: score ≥ 8; possible CUD: score ≥ 13)

* p < 0.05

** p < 0.01

**Table 4:** Group differences in sGFAP *Z *scores by psychometric cutoffs (Welch’s *t *tests)

Instrument	< cutoff (M, SD, *n*)	≥ cutoff (M, SD, *n*)	*t*(df)	*p* (2-sided)	Hedges’ *g* [95% CI]
AUDIT-C	−0.34, 1.10, 197	−0.48, 1.03, 134	0.20 (297.7)	0.233	0.13 [−0.09, 0.35]
ERIraos	−0.32, 1.07, 225	−0.57, 1.07, 106	2.00 (205.0)	0.047*	0.24 [0.00, 0.47]
PHQ-9	−0.39, 1.08, 295	−0.47, 1.07, 36	0.45 (44.1)	0.656	0.08 [−0.27, 0.42]
GAD-7	−0.40, 1.06, 315	−0.41, 1.37, 16	0.04 (15.9)	0.972	0.01 [−0.49, 0.51]
ASRS	−0.39, 1.02, 287	−0.41, 1.38, 44	0.10 (50.4)	0.924	0.02 [−0.30, 0.34]
CUDIT-R hazardous use	−0.44, 1.10, 78	−0.38, 1.07, 253	−0.42 (124.5)	0.673	−0.06 [−0.31, 0.20]
CUDIT-R possible CUD	−0.37, 1.07, 224	−0.46, 1.08, 107	0.73 (208.3)	0.467	0.09 [−0.14, 0.32]

*M* mean, *SD* standard deviation, *n* sample size, *CI* confidence interval, *AUDIT-C* Alcohol Use Disorder Identification Test–Consumption, *ERIraos* Early Recognition Inventory for Psychosis, *PHQ-9* Patient Health Questionnaire for Depression, *GAD-7* Generalized Anxiety Disorder Scale-7, *ASRS* Adult ADHD Self-Report Scale, *CUD* cannabis use disorder, *CUDIT-R* Cannabis Use Disorder Identification Test–Revised (hazardous use: score ≥ 8; possible CUD: score ≥ 13)

* p < 0.05

**Table 5:** Linear regression model with sNfL *Z* scores as dependent variable (*n* = 331)

Variable	*B*	SE	*t*	*p*	95% CI
(Constant)	−0.109	0.098	−1.114	0.266	−0.301 to 0.083
CUDIT-R category	0.167	0.074	2.253	0.025*	0.021 to 0.314

*R*^2^ = 0.015; adjusted *I*^2^ = 0.012; *n* = 331. Categories: below cutoffs (0–7 points), hazardous use (8–12 points), and possible CUD (13–32 points)

*CUDIT-R* Cannabis Use Disorder Identification Test–Revised

* p < 0.05

### Bivariate analyses: correlation of neurofilaments with psychometric instrument scores

sNfL levels showed a positive correlation with ERIraos and CUDIT-R scores. sGFAP levels were negatively associated with the ERIraos score (Table 6).

**Table 6:** Spearman rank correlation of sNfL/sGFAP *Z* scores with psychometric instruments scores (*n* = 331)

sNfL	AUDIT-C	ERIraos	PHQ-9	GAD-7	ASRS	CUDIT-R
*ρ*	−0.097	0.146	−0.027	−0.022	0.041	0.118
*p*-value	0.079	0.008**	0.629	0.684	0.461	0.032*
sGFAP	AUDIT-C	ERIraos	PHQ-9	GAD-7	ASRS	CUDIT-R
*ρ*	−0.003	−0.114	−0.065	−0.040	−0.042	−0.010
*p*-value	0.951	0.038*	0.235	0.466	0.447	0.849

*ρ* Spearman’s rho, *AUDIT-C* Alcohol Use Disorder Identification Test–Consumption, *ERIraos* Early Recognition Inventory for Psychosis, *PHQ-9* Patient Health Questionnaire for Depression, *GAD-7* Generalised Anxiety Disorder Scale-7, *ASRS* Adult ADHD Self-Report Scale, *CUDIT-R* Cannabis Use Disorder Identification Test–Revised

* p < 0.05

** p < 0.01

### Bivariate analyses between CUDIT-R items neurofilament levels

**Fig. 2:**
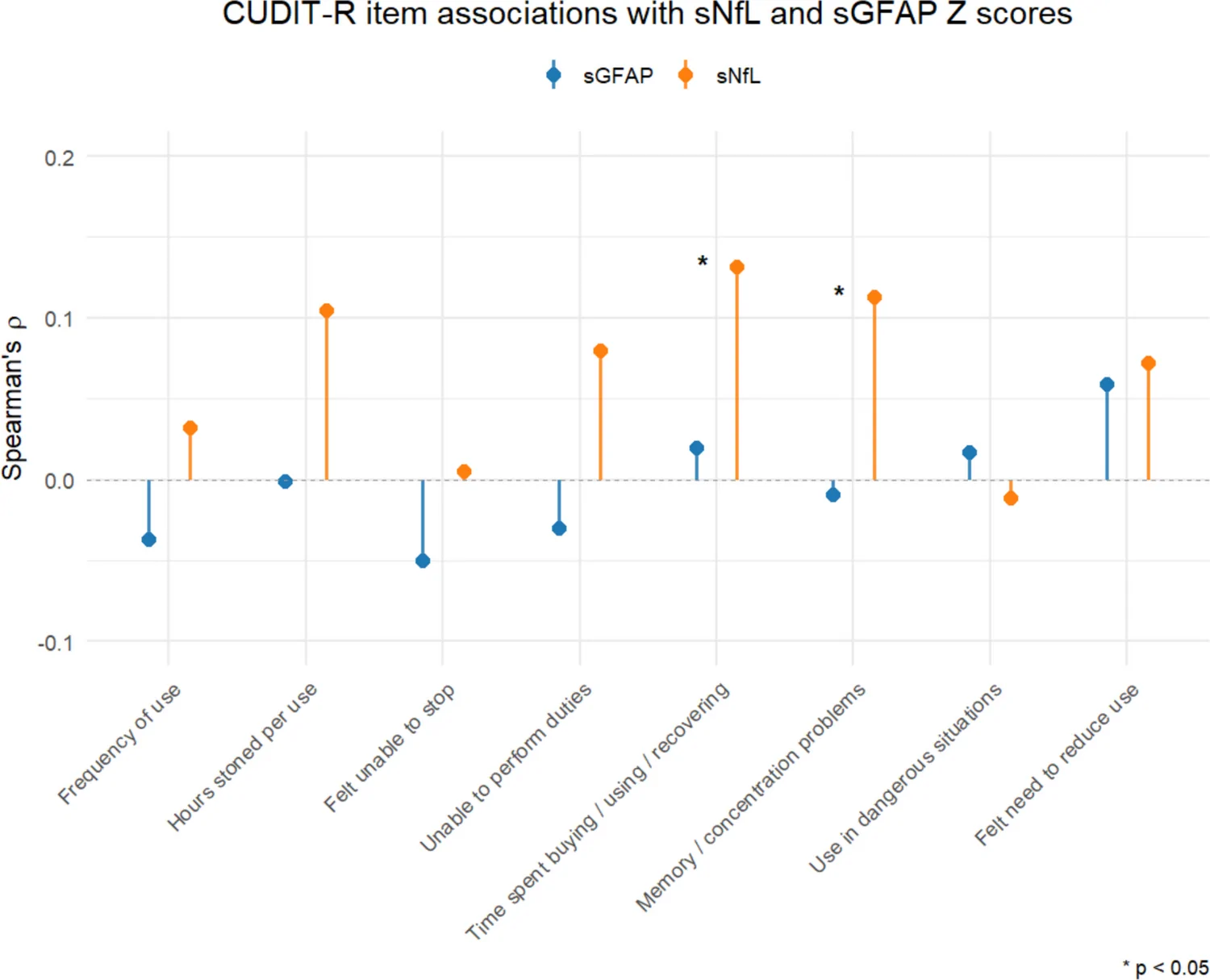
Correlation of NfL/GFAP *Z* scores with CUDIT-R items (*n* = 331). CUDIT-R, Cannabis Use Disorder Identification Test–Revised; sNfL, serum neurofilament light chain; sGFAP, serum glial fibrillary acidic protein; ρ, Spearman’s rho

Spearman rank correlations were used to test associations between specific CUDIT-R items and sNfL/sGFAP levels. sNfL *Z* scores were positively correlated with the CUDIT-R item on memory/concentration problems (*ρ*(329) = 0.11, *p* = 0.04) and the item inquiring about time spent acquiring cannabis (*ρ*(329) = 0.13, *p* = 0.02) as determined by Spearman rank correlation tests (Fig. 2).

### Bivariate analyses between self-reported cannabis use and neurofilament levels

No relationship was found between sNfL and self-reported THC cannabis use days in the past 30 days (*r*(318) = 0.04, *p* = 0.43), or sNfL and average amount of THC cannabis used in grammes (*r*(319) = 0.07, *p* = 0.23). Likewise, there was no relationship between sGFAP and self-reported THC cannabis use days in the past 30 days (*r*(318) = −0.04, *p* = 0.43), or sGFAP and average amount of THC cannabis used in grammes (*r*(319) = 0.00, *p* = 0.99). As a sensitivity analysis, we repeated the correlation after excluding participants with implausible values on self-reported amount of THC cannabis used (*Z* > 3, *n* = 9 excluded). Results remained consistent (Supplemental Material, Result A1).

### Multivariate analyses

**Fig. 3:**
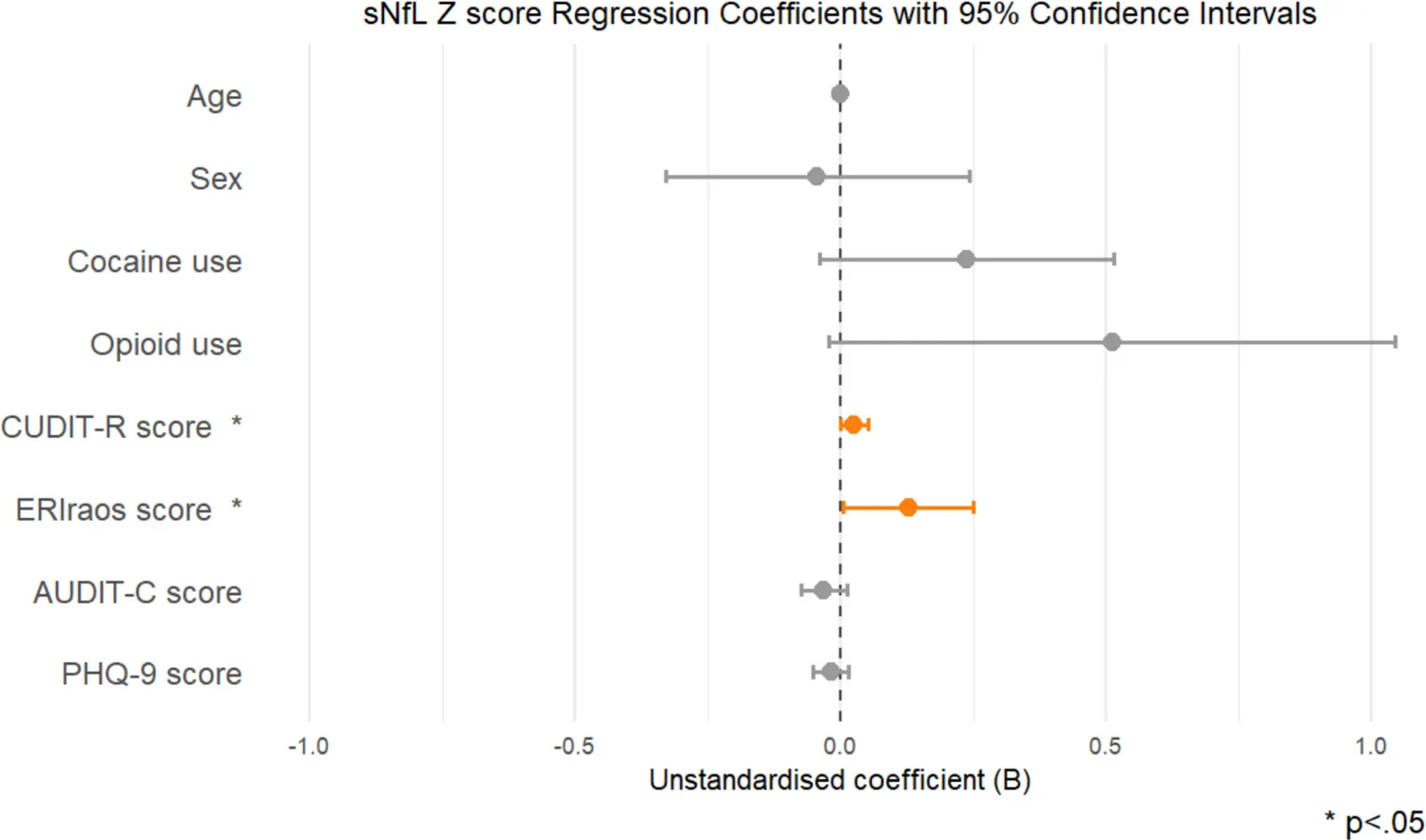
Multiple linear regression model with sNfL *Z* scores as the dependent variable (*n* = 331). Sex: male was the reference category. CUDIT-R, Cannabis Use Disorder Identification Test–Revised; ERIraos, Early Recognition Inventory for Psychosis; AUDIT-C, Alcohol Use Disorder Identification Test–Consumption; PHQ-9, Patient Health Questionnaire for Depression

For sNfL, cocaine and opioid use were entered as binary confounders due to their reported association with altered NfL levels [23, 24, 39]. CUDIT-R, ERIraos, and AUDIT-C scores were entered as continuous variables. Although the association between PHQ-9 score and sNfL did not reach statistical significance in bivariate analyses, it was also included as a confounder due to the known link between major depressive disorder and NfL elevation [20]. The model is provided in Table 7 and Fig. 3. A sensitivity analysis adjusting for time delay (number of days between blood sampling and questionnaire assessment) as a covariate yielded comparable results (Supplemental Material, Table A2).

**Table 7:** Multiple linear regression model with sNfL *Z* scores as dependent variable

Variable	*B*	SE	*t*	*p*	95% CI
(Constant)	−0.082	0.250	−0.328	0.743	−0.573 to 0.410
Age	−0.001	0.005	−0.302	0.763	−0.011 to 0.008
Sex^a^	−0.044	0.145	−0.301	0.763	−0.329 to 0.242
Cocaine use	0.237	0.145	1.693	0.092	−0.039 to 0.514
Opioid use	0.512	0.271	1.889	0.060	−0.021 to 1.045
CUDIT-R score	0.026	0.013	2.032	0.043*	0.001 to 0.052
ERIraos score	0.128	0.062	2.062	0.040*	0.006 to 0.250
AUDIT-C score	−0.031	0.022	−1.400	0.163	−0.074 to 0.013
PHQ-9 score	−0.018	0.017	−1.081	0.280	−0.051 to 0.015

*R*^2^ = 0.063; adjusted *R*^2^ = 0.040; *n* = 331; VIFs < 1.3 for all variables

^a^Male was the reference category

*CUDIT-R* Cannabis Use Disorder Identification Test–Revised, *ERIraos* Early Recognition Inventory for Psychosis, *AUDIT-C* Alcohol Use Disorder Identification Test–Consumption, *PHQ-9* Patient Health Questionnaire for Depression

* p < 0.05

For sGFAP, CUDIT-R and ERIraos scores were entered as continuous variables. PHQ-9 scores were included in the model due to the link between GFAP and unipolar depression in the medical literature [21]. The model is provided in the Supplemental Material (Table A3).

## Discussion

This is the first study to investigate the association between cannabis use and sNfL and sGFAP levels. There was no association between self-reported cannabis use amount or frequency and sNfL or sGFAP levels. We found that the severity of cannabis use (CUDIT-R) and psychotic symptoms (ERIraos) were positively correlated with sNfL levels. Additionally, participants with hazardous cannabis use had higher mean sNfL levels. The specific association remained significant controlling for other factors known to influence sNfL levels, such as alcohol and cocaine use. Furthermore, mean sGFAP levels were lower in our sample compared to the general population, and specifically lower in participants with psychotic symptoms. However, when controlling for age, sex, CUDIT-R score, and depressive symptoms, this association was no longer present.

NfL has previously been studied in other substance use populations, specifically alcohol-, opioid-, and cocaine-dependent individuals [23, 24, 39]. These studies found elevated serum or cerebrospinal fluid NfL levels in individuals using the respective substance compared to healthy controls but did not consider NfL’s dependence on age and BMI [19]. Our study accounted for this by calculating sNfL level *Z* scores from a large reference database. In accordance with the findings reported in these studies, we show a similar link between NfL elevations and cannabis use severity in regular cannabis users. This difference was found when comparing hazardous cannabis users to those below the CUDIT-R cutoffs but not when comparing hazardous cannabis users to those with possible CUD. This may indicate a threshold effect of THC use on neuroaxonal structures. However, this association should be interpreted carefully as overall mean NfL levels in our sample did not differ from the reference population. In addition, no relationship between self-reported THC cannabis use frequency or amount and sNfL levels was found. This discrepancy could indicate that potential neurotoxic effects are more closely linked to patterns of problematic use, as captured by the CUDIT-R, than to self-reported consumption amount alone. Notably, our sample comprised occasional, regular, and daily cannabis users. This heterogeneity may have masked potential threshold effects, as changes in NfL/GFAP levels might only emerge with sustained, heavy use as compared to lighter use patterns.

Several studies link chronic cannabis use to cognitive impairment. Cannabis use has been suggested to have neurotoxic effects, particularly on the adolescent brain [40, 41]. In rodent models, THC exposure increases neuronal oxidative stress, induces mitochondrial dysfunction, and is linked to altered synaptic transmission [42, 43]. Notably, these models often use extremely high doses of THC or synthetic cannabinoid–receptor agonists, limiting comparability to human studies. Still, our finding that the association between NfL and the CUDIT-R is largely driven by the item “memory/concentration problems” is of particular interest. It may reflect potential neurotoxic effects through neuroaxonal injury caused by THC exposure. If future studies replicate the association, this item may serve as an indicator of neuronal damage. However, as causal mechanisms cannot be inferred from this cross-sectional investigation, participants with a preexisting vulnerability not captured by our study may be more likely to develop heavier cannabis use. Additionally, we found a link between sNfL levels and the CUDIT-R item “time spent buying/using/recovering.” As this item is somewhat unspecific, it is unclear which domain of cannabis use was captured exactly. Furthermore, most participants in this study likely used cannabis concurrently with tobacco, which is common practice in Europe. Previous research demonstrated an association between NfL levels and tobacco use [44], which may have confounded our analysis. Future research would ideally be longitudinal and investigate the association of NfL with self-reported as well as objective measures of concentration, memory, and other cognitive functions.

Interestingly, psychotic symptoms as assessed by the ERIraos were positively associated with sNfL and negatively associated with sGFAP. These findings are partly in line with previous research, showing that sNfL is associated with symptoms of schizophrenia [45]. For GFAP, there is evidence for glial cell loss and the resulting reduced astroglial activity in individuals suffering from schizophrenia [46]. However, other research investigating GFAP levels in patients with schizophrenia found no difference or elevated levels [47, 48]. When we controlled for other variables of interest in multiple linear regression, the association between psychotic symptoms and sGFAP did not persist. The overall lower mean sGFAP levels further complicate biological interpretation of our results and do not align with the neurotoxicity hypothesis, underlining that further longitudinal research with objective measurements of THC intake is needed.

Taken together, our findings provide preliminary evidence that regular recreational cannabis use may be associated with subtle neuroaxonal alterations as reflected by increased sNfL levels, particularly among individuals with more severe CUD symptoms as assessed through the CUDIT-R. However, these observed effects were small and within a normative biomarker range in a relatively young sample, limiting support for a neurotoxicity interpretation. sGFAP levels were overall lower and no association remained significant after adjusting for covariates, which may argue against astroglial injury in our sample. Future longitudinal research combining NfL and GFAP quantification with objective measures of cannabis exposure is needed to clarify the temporal relationship between cannabis use and these biomarkers.

### Limitations

This study has several limitations. First, the cross-sectional design precludes conclusions about causality between cannabis use and neurofilament alterations. Second, although *Z* scores were derived from a large reference population and adjusted for age, BMI, and (for GFAP) sex, residual confounding from unmeasured factors such as sleep, diet, physical activity, or co-occurring mental disorders cannot be ruled out. This also applies to potential health issues in our sample that may present an additional confounding factor to NfL/GFAP measurements such as neurological disorders or a history of head injuries, which were not collected by the parent study. Similarly, the presence of noncannabis substance use disorders such as cocaine use disorder and opioid use disorder may present a confounding factor that was not assessed by the parent study. As this study compared regular cannabis users to a normative reference sample, unmeasured differences between the groups, particularly regarding substance use patterns and psychiatric comorbidities, likely exist and could have influenced any observed deviations. Third, substance use and psychiatric symptoms were self-reported, introducing potential recall and reporting biases. Fourth, in some participants, the delay of up to 16 weeks between blood sampling and questionnaire completion may have led to temporal mismatch between biomarker levels and self-reported symptoms. There is a possibility that this introduces a relevant bias to our results. However, the sample consisted of chronic and regular cannabis users. Substantial changes in use behaviour during this interval are unlikely, mitigating the potential impact of this limitation. Fourth, covariate selection differed between NfL and GFAP models. While NfL covariates were informed by established literature in addition to associations found in bivariate analyses, the limited evidence on substance use and GFAP levels meant that covariate selection relied primarily on sample-specific bivariate associations. This inconsistency in covariate control may limit direct comparability between the two biomarker models and raises the potential for residual confounding in the GFAP analyses. An additional concern is the potential impact of tobacco use on sNfL and sGFAP, which we could not control for due to a lack of granular assessment in the parent study. Additionally, the parent study did not collect data on the specific THC/CBD content of cannabis used by participants. Hence, this information could not be included in the present analyses. Finally, beyond the general limitations of self-reported data on cannabis use amount and frequency (such as recall bias, imprecise quantification, and missing data), a small number of participants provided implausible values. We accounted for this by conducting a sensitivity analysis, excluding extreme values which yielded consistent results.

## Supplementary Information

Below is the link to the electronic supplementary material.

Supplementary file1 (DOCX 27 kb)

## Data Availability

The datasets generated and analysed during the current study are not publicly available due to privacy reasons but are available from the corresponding author on reasonable request.
