# Supplementary material for: NfL and GFAP quantification and associations with mental health in recreational cannabis users—Results from the Swiss study on recreational cannabis access via pharmacies
Source: Mol Neurobiol. 2026 Mar 13;63(1):497. doi: 10.1007/s12035-026-05798-9 (PMC13649837; doi:10.1007/s12035-026-05798-9)
Supplement: Supplementary file 1 [file 12035_2026_5798_MOESM1_ESM.docx]

**NfL and GFAP quantification and associations with mental health in recreational cannabis users - Results from the Swiss study on recreational cannabis access via pharmacies**

Maximilian Meyer^1,*^, C. Felix Mosandl^1^, Adrian Guessoum^1^, Oliver Herrmann^1^, Lavinia Baltes-Flückiger^2^, Eva-Maria Pichler^2^, Amar Zadic^3^, Aleksandra Maleska Maceski^3^, Juan Francisco Vilchez Gomez^3^, Kenneth M. Dürsteler^1^, Felix Müller^1^, Undine E. Lang^1^, Pascal Benkert^3^, Jens Kuhle^3^, Marc Walter^2,§^, Marc Vogel^1,§^

1 University of Basel Psychiatric Clinics, University of Basel, Basel, Switzerland

2 Psychiatric Services Aargau, Windisch, Switzerland

3 Multiple Sclerosis Centre and Research Center for Clinical Neuroimmunology and Neuroscience (RC2NB), Departments of Clinical Research, Neurology and Biomedicine, University Hospital and University of Basel, Basel, Switzerland

* Corresponding author: Maximilian Meyer ([maximilian.meyer@upk.ch](mailto:maximilian.meyer@upk.ch))

§ MW and MV contributed equally to this work and share last authorship

Journal: Molecular neurobiology

*Table A1. Sociodemographic, medical, and psychometric characteristics by CUDIT-R group (n=331)*

|  |  | CUDIT-R | | |
| --- | --- | --- | --- | --- |
|  |  | below cut-off (n=78) | hazardous use (n=146) | possible CUD (n=107) |
| Age (M, SD) |  | 36.5, 10.1 | 35.8, 11.7 | 34.3, 12.4 |
|  |  |  |  |  |
| Sex (n, %) | male | 66, 84.6 | 117, 80.1 | 88, 82.2 |
|  | female | 12, 15.4 | 29, 19.9 | 19, 17.8 |
|  |  |  |  |  |
| Substance use in the past 6 months (n, %) | Alcohol | 72, 92.3 | 131, 89.7 | 97, 90.7 |
|  | Hallucinogens | 13, 16.7 | 29, 19.9 | 26, 24.3 |
|  | Amphetamines | 10, 12.8 | 30, 20.5 | 28, 26.2 |
|  | Cocaine | 9, 11.5 | 36, 24.7 | 22, 20.6 |
|  | Benzodiazepines | 4, 5.1 | 11, 7.5 | 16, 15.0 |
|  | Opioids | 3, 3.8 | 4, 2.7 | 8, 7.5 |
|  | Glue / solvent | 0, 0 | 6, 4.1 | 1, 0.9 |
|  |  |  |  |  |
| Cannabis use in the past 30 days (n, %) | CBD flowers | 27, 34.6 | 53, 36.3 | 38, 35.5 |
|  | CBD hashish | 5, 6.4 | 11, 7.5 | 9, 8.4 |
|  | CBD concentrate | 4, 5.1 | 13, 8.9 | 7, 6.5 |
|  | THC flowers | 67, 85.9 | 137, 93.8 | 98, 91.6 |
|  | THC hashish | 41, 52.6 | 83, 56.8 | 70, 65.4 |
|  | THC concentrate | 6, 7.7 | 28, 19.2 | 20, 18.7 |
|  |  |  |  |  |
| Days of THC cannabis use in the past 30 days (M, SD) |  | 9.3, 8.6 | 18.4, 10.6 | 24.1, 8.5 |
| Average amount of THC cannabis used per day in grams (M, SD) |  | 0.5, 0.7 | 1.6, 3.2 | 1.8, 2.7 |
|  |  |  |  |  |
| PHQ-9 | score (M, SD) | 3.8, 3.5 | 5.1, 3.5 | 6.1, 3.6 |
|  | above cut-off (n, %) | 4, 5.1 | 16, 11.0 | 16, 15.0 |
| GAD-7 | score (M, SD) | 2.8, 3.2 | 3.4, 3.1 | 4.5, 3.2 |
|  | above cut-off (n, %) | 4, 5.1 | 6, 4.1 | 6, 5.6 |
| ASRS | score (M, SD) | 0.8, 1.2 | 1.5, 1.4 | 2.3, 1.5 |
|  | above cut-off (n, %) | 3, 3.8 | 16, 11.0 | 25, 23.4 |
| ERIraos | score (M, SD) | 0.2, 0.6 | 0.5, 0.8 | 0.7, 1.2 |
|  | above cut-off (n, %) | 13, 16.7 | 49, 33.6 | 44, 41.1 |
| AUDIT-C | score (M, SD) | 3.9, 2.1 | 3.7, 2.7 | 3.8, 2.6 |
|  | above cut-off (n, %) | 34, 43.6 | 57, 39.0 | 43, 40.2 |

M: mean; SD: standard deviation; CUD: cannabis use disorder; CUDIT-R: Cannabis Use Disorder Identification Test – Revised; below cut-off: CUDIT-R score < 8; hazardous use: CUDIT-R score between 8 and 12; possible CUD: CUDIT-R score ≥ 13; CBD: cannabidiol; THC: Δ^9^-tetrahydrocannabinol; PHQ-9: Patient Health Questionnaire for Depression; GAD-7: Generalized Anxiety Disorder Scale-7; ASRS: Adult ADHD Self-Report Scale; ERIraos: Early Recognition Inventory for Psychosis; AUDIT-C: Alcohol Use Disorder Identification Test – Consumption;

*Result A1. Sensitivity analysis of the association between self-reported cannabis use and neurofilament levels*

Participants with implausible values on self-reported amount of THC cannabis used (Z > 3, n = 9) were excluded. No relationship was found between sNfL and THC cannabis use days in the past 30 days (r(309) = .06, p = .33). No relationship was found between sNfL and average amount of THC cannabis in grams (r(310) = .09, p = .11). No relationship was found between sGFAP and THC cannabis use days in the past 30 days (r(309) = -.04, p = .51). No relationship was found between sGFAP and average amount of THC cannabis in grams (r(310) = -.01, p = .82).

*Table A2. Sensitivity analysis of the multiple linear regression model with sNfL Z scores as dependent variable controlling for time delay*

| Variable | B | SE | t | p | 95% CI |
| --- | --- | --- | --- | --- | --- |
| (Constant) | -.004 | .312 | -.013 | .990 | -.618 – .610 |
| Age | -.001 | .005 | -.283 | .778 | -.011 – .008 |
| Sex^a^ | -.045 | .145 | -.310 | .756 | -.331 – .241 |
| Cocaine use | .241 | .141 | 1.712 | .088 | -.036 – .518 |
| Opioid use | .511 | .271 | 1.884 | .060 | -.023 – 1.045 |
| CUDIT-R score | .026 | .013 | 2.026 | .044* | .001 – .052 |
| ERIraos score | .127 | .062 | 2.048 | .041* | .005 – .250 |
| AUDIT-C score | -.032 | .022 | -1.431 | .153 | -.075 – .012 |
| PHQ-9 score | -.018 | .017 | -1.104 | .270 | -.051 – .014 |
| Time delay | -.001 | .002 | -.417 | .677 | -.005 – .003 |

R^2^ = 0.072; adjusted R^2^ = 0.045; n = 331; VIFs < 1.3 for all variables. a: male was the reference category. CUDIT-R: Cannabis Use Disorder Identification Test – Revised; ERIraos: Early Recognition Inventory for Psychosis; AUDIT-C: Alcohol Use Disorder Identification Test – Consumption; PHQ-9: Patient Health Questionnaire for Depression; Time delay: number of days between blood sampling and questionnaire assessment;

*Table A3. Multiple linear regression model with sGFAP Z scores as dependent variable*

| Variable | B | SE | t | p | 95% CI |
| --- | --- | --- | --- | --- | --- |
| (Constant) | -.129 | .251 | -.512 | .609 | -.622 – .365 |
| Age | -.006 | .005 | -1.165 | .245 | -.016 – .004 |
| Sex^a^ | -.213 | .155 | -1.370 | .171 | -.519 – .093 |
| CUDIT-R score | .001 | .014 | .054 | .957 | -0.027 – .028 |
| ERIraos score | -.058 | .068 | -.860 | .391 | -.191 – .075 |
| PHQ-9 score | .001 | .018 | .048 | .962 | -0.034 – .036 |

R^2^ = 0.012; adjusted R^2^ = -0.003; n = 331; VIFs < 1.2 for all variables. a: male was the reference category. CUDIT-R: Cannabis Use Disorder Identification Test – Revised; ERIraos: Early Recognition Inventory for Psychosis; PHQ-9: Patient Health Questionnaire for Depression;
